# SRFACS: A secure and robust framework for anonymous communication systems

**DOI:** 10.1371/journal.pone.0312817

**Published:** 2024-12-02

**Authors:** Daxin Zhu, Jiazhi Tu, Danlin Cai, Tianyu Jiang, Jianbing Xiahou, Yusi Chen, Chao Liu

**Affiliations:** 1 School of Mathematics and Computer Science, Fujian Provincial Key Laboratory of Data-Intensive Computing, Fujian University Laboratory of Intelligent Computing and Information Processing, Quanzhou Normal University, Quanzhou, China; 2 Fuzhou University, Fuzhou, China; 3 Xiamen University, Xiamen, China; 4 Fujian Normal University, Fuzhou, China; 5 Yangtze Delta Region Institute of Tsinghua University, Zhejiang, Jiaxing, China; Jinan University, CHINA

## Abstract

Anonymous communication is crucial for preserving user privacy in various applications, such as anonymous browsing, secure online payments, and electronic voting. However, current systems face significant challenges related to robustness, fault tolerance, and efficient communication management. This paper introduces SRFACS (Secure and Robust Framework for Anonymous Communication Systems), designed to address these issues by integrating advanced cryptographic techniques with a structured communication framework. Traditional anonymous communication systems usually lack sufficient fault tolerance, making them vulnerable to node failures, especially in asynchronous environments. In order to overcome these limitations and ensure that the system can keep running in the event of node failure (especially in an asynchronous environment), SRFACS integrates an improved asynchronous Byzantine Fault Tolerant (ABFT) protocol. We use this protocol to expand the traditional single communication node into a structured node group, and handle up to (*n* − 1)/3 faulty nodes in these groups, so as to enhance fault tolerance and maintain continuous operation. To efficiently manage inter-group communication, SRFACS utilizes leader nodes to coordinate and streamline the communication processes. To mitigate the risks associated with leader failures, we have implemented an efficient leader change protocol that promptly replaces defective leaders, ensuring uninterrupted system performance. Additionally, to prevent erroneous leader actions from compromising the system, we have introduced an advanced multi-signature scheme. This approach secures communication by requiring multiple signatures for verification processes. Furthermore, we have implemented a reputation incentive mechanism to encourage nodes to maintain optimal performance and avoid malicious behavior. This mechanism evaluates nodes according to their past activities and reliability to achieve dynamic updates of SRFACS node groups. We have conducted rigorous security analyses and component performance evaluations of SRFACS, further confirming its potential as a promising secure communication solution.

## Introduction

The internet has extensive applications in our daily lives. We use it almost every day for various activities, such as transactions and communication. However, when the internet environment is untrustworthy, it poses significant threats and attacks on user privacy and communication security. Our communication messages can be intercepted or subjected to traffic analysis attacks, leading to the leakage of user privacy. Some attackers may sell this private information, posing a severe threat to user privacy and security. To mitigate these risks, many users prefer to use anonymous communication [1, 2] to hide their real communication relationships, thus achieving privacy protection for user identities. This ensures that eavesdroppers cannot directly know or indirectly infer the communication relationships and identities of the parties involved. Anonymous communication protects the identities of both the initiator and the recipient of the communication, as well as the relationship between them.

Currently, anonymous communication is widely used in various applications and platforms. For example, Tor [3] is a popular platform that enables anonymous browsing and communication by routing internet traffic through a network of volunteer-operated servers, concealing users’ locations and usage from surveillance and traffic analysis. Similarly, services like I2P [4] and Freenet [5] provide anonymous networking solutions to protect user privacy. In specific applications, anonymous communication is crucial in areas such as anonymous emails (e.g., ProtonMail), electronic online voting, and online payments [6].

Recent advancements in anonymous communication systems have demonstrated excellent performance and robustness. Systems like cMix [7], Atom [8], Stadium [9], and XRD [10] have employed various innovative techniques to enhance their reliability and efficiency. These systems rely on different methods to achieve robustness, such as random partial checking and trap message methods used in cMix, non-interactive zero-knowledge proofs (NIZK) and trap message methods in Atom, and NIZK in Stadium and XRD.

In a **random partial checking (RPC)** mix network, mix nodes produce strong probabilistic evidence of their correct operation by partially revealing their input/output batch relation. However, RPC provides only a probabilistic guarantee, and a compromised mix node might not be detected. A **trap message** tests the reliability of a mix network by verifying if the message appears correctly on the output side. While this method can indicate the presence of a malicious node, it cannot identify the specific source of the failure and may require a high number of trap messages for adequate probabilistic guarantees. **Zero-knowledge proofs** allow nodes in the cascade to prove, without revealing any information, that they performed their tasks correctly. These proofs can be published after real-time operations, thus not affecting the network’s performance. Zero-knowledge proofs are considered highly effective for achieving robustness in mix networks because they allow for easy detection of the failure source and verification of proper operation by any party. Although they may require significant computation and bandwidth, systems like Atom, Stadium, and XRD show excellent performance when incorporating NIZK in their architectures.

Despite these advancements, traditional anonymous communication systems still encounter significant limitations. They are often unable to tolerate faulty nodes and can only detect failures when messages are transmitted from senders to receivers using methods such as zero-knowledge proofs, trap messages, or random partial checking. Furthermore, these systems usually lack mechanisms to penalize detected faults, leaving them vulnerable to repeated attacks by malicious nodes.

This paper proposes SRFACS, a novel anonymous communication system that integrates several key components: an Asynchronous Byzantine Fault Tolerance (ABFT) protocol, a Multi-Signature algorithm (MSP), a leader change protocol, and a reputation-based incentive mechanism. These components work together to enhance the robustness, efficiency, and security [11, 12]of the system. SRFACS addresses the limitations of traditional systems by ensuring fault tolerance [13], timely detection and replacement of faulty leaders, and incentivizing high-quality nodes through a reputation-based mechanism.

### Efficiency of SRFACS

The proposed SRFACS framework integrates several key components, including the Asynchronous Byzantine Fault Tolerance (ABFT) protocol, a Multi-Signature Process (MSP), a Leader Change Protocol, and a Reputation Incentive Mechanism, each of which impacts both time and communication complexities.

The time complexity of the SRFACS framework primarily depends on the components used for communication, signature aggregation, consensus, and reputation calculation. The ABFT protocol, which is central to the fault tolerance mechanism, has a time complexity of *O*(*N*) in both optimal and worst-case scenarios, as it can tolerate up to (*n* − 1)/3 faulty nodes, ensuring robust performance. The Multi-Signature Process (MSP) adds computational overhead during key generation, signature creation, and aggregation. The MSP signature generation for each node occurs in *O*(1) time, while the aggregation of signatures, requiring communication between nodes, operates in *O*(*N*). The Reputation Incentive Mechanism involves calculating reputation scores based on several factors (participation, correctness, and bandwidth), which incurs an additional complexity of *O*(*N*) for each epoch, as the system must evaluate all participating nodes.

In terms of communication complexity, the SRFACS system performs efficiently. The ABFT protocol requires *O*(*N*) messages in the best case, scaling linearly with the number of nodes. The Multi-Signature Process contributes by requiring the broadcasting of signatures and the verification of aggregated signatures, which adds to the communication overhead but still maintains a linear complexity of *O*(*N*). The Reputation Incentive Mechanism also introduces communication overhead for gathering node performance data and updating reputation scores. This operation occurs periodically (per epoch), and thus has a communication complexity of *O*(*N*). Overall, the communication complexity of the proposed method in both optimal and worst-case scenarios is *O*(*N*), as shown in Table 1.

**Table 1:** Time and communication complexity of SRFACS components and overall system.

Component	Time Complexity	Communication Complexity
ABFT Protocol	*O*(*N*)	*O*(*N*)
MSP (Signature Generation)	*O*(1)	*O*(*N*)
MSP (Signature Aggregation)	*O*(*N*)	*O*(*N*)
Leader Change Protocol	*O*(*N*)	*O*(*N*)
Reputation Incentive Mechanism	*O*(*N*)	*O*(*N*)
**SRFACS (Overall)**	*O*(*N*)	*O*(*N*)

### Related work

#### Anonymous communication

The Tor network is extensively utilized for anonymous communication; however, its present low-latency architecture makes it highly vulnerable to traffic analysis. This susceptibility has been shown through both theoretical research and empirical evidence [14, 15].

Mixing networks (mixnets) [2] bolster protection against traffic analysis by incorporating higher latency through multiple hops and blending messages at one or more trusted nodes. Over the past forty years, various mixnet-inspired protocols [7, 16] have been devised to combat traffic analysis attacks. Despite their effectiveness, these protocols often involve significant latency overheads, typically lasting several seconds, which are impractical for applications such as browsing, messaging, and video calls. Additionally, mixnets are inherently fragile; the failure or crash of a single node can result in message loss.

Layered encryption is also a fundamental concept in onion routing. In this technique, the sender selects a random path of “somewhat trusted” routers and encrypts the packet in multiple layers, each corresponding to a router along the path. Each router removes one layer of encryption, with the final router decrypting the innermost layer and delivering the content to the intended recipient. The Tor network [3], the most widely used anonymous communication system, relies on onion routing as its core mechanism.

Dissent [17, 18] introduces semi-trusted servers to make dining cryptographers networks (DCnets) [19] practical on a reasonable scale. This approach ensures anonymity as long as at least one server is honest. Additionally, Dissent offers accountability, a feature often overlooked in most anonymous communication protocols. However, Dissent depends on the assumption that all members remain connected and send correctly signed messages throughout each round. Moreover, excluding a single disruptor is time-consuming, requiring *O*(*N*) time.

In [20], a novel approach to creating a coercion-resistant voting system is detailed through the introduction of VoteXX. This system features a unique mechanism where votes can be nullified by designated trusted agents known as “hedgehogs.” In this scheme, each voter is assigned two sets of public-private key pairs. Without disclosing their private keys, voters register their public keys with the election authority. Additionally, voters have the option to share their keys with one or more hedgehogs, adding an extra layer of security and ensuring the integrity of the voting process.

Secure multi-party computation (MPC) [21] has been suggested as a method to implement commit-and-reveal functionality while ensuring anonymous input disclosure, a concept also known as anonymous committed broadcast (ACB). This approach [22] anonymizes inputs by randomly shuffling them efficiently. Honest-majority MPC protocols are preferred in this context because they guarantee the correctness of the output as long as the majority of participants are honest.

#### Asynchronous BFT

Among Byzantine Fault Tolerant (BFT) protocols, completely asynchronous BFT protocols have gained renewed attention due to their inherent robustness. Once considered primarily theoretical, recent implementations of asynchronous BFT systems—such as HALE [23], Dumbo [24], and FACOS [25]—have demonstrated performance levels comparable to their partially synchronous counterparts. Asynchronous Byzantine Fault Tolerant (BFT) protocols offer significant advantages, including robustness to unpredictable network conditions, improved security by being less vulnerable to timing attacks, higher resilience to network partitioning and delays, better scalability for large and geographically distributed systems, and greater flexibility in adapting to various network environments. These qualities make asynchronous BFT protocols particularly well-suited for decentralized and distributed systems where network reliability and timing cannot be guaranteed.

Dashing and Star propose a BFT mechanism that utilizes weak certificates, enhancing the protocol’s resilience while maintaining efficiency under Byzantine conditions [26]. Similarly, FIN introduces a practical, signature-free asynchronous common subset that operates in constant time, offering a significant improvement in consensus protocols by removing the reliance on digital signatures [27]. Sharding blockchains have also seen innovations aimed at improving cross-shard consensus. CHERUBIM introduces a quadruple pipelined two-phase commit mechanism for secure and highly parallel cross-shard consensus [28], while another protocol proposes a flexible sharding mechanism based on cross-shard BFT to enhance the scalability and security of blockchain systems [29].

#### Trust and reputation systems

In the realm of federated learning and digital twin systems, trust evaluation is crucial. The TFL-DT scheme offers a novel approach to trust evaluation within federated learning, specifically tailored for mobile networks, ensuring that digital twins can be effectively managed and secured [30]. Furthermore, PPRU introduces a privacy-preserving reputation updating scheme for cloud-assisted vehicular networks, addressing the critical need for privacy and trust in such dynamic environments [31].

### Issues

Anonymous communication systems face several critical issues that undermine their performance and security.

#### Deficient fault tolerance mechanisms

Most existing anonymous communication protocols suffer from inadequate fault tolerance, which can have severe implications for system efficiency and reliability. In these systems, a single point of failure—such as a malfunctioning node or a network partition—can lead to significant disruptions, including service outages or data loss. This lack of fault tolerance means that the system may be unable to recover quickly or gracefully from failures, resulting in degraded performance or complete system collapse. Effective fault tolerance is essential to ensure that the system can maintain its functionality and performance even when some nodes fail or exhibit faulty behavior. This involves implementing mechanisms to handle node failures, such as redundancy, error detection, and recovery strategies, to minimize the impact of individual failures and enhance the overall robustness of the communication protocol. Without such measures, anonymous communication systems are vulnerable to reliability issues, which can undermine user trust and system effectiveness.

Additionally, the absence of a leader mechanism within node groups further exacerbates fault tolerance issues. A leader node is crucial for coordinating and managing communication, and without it, systems can experience inefficient communication processes and increased vulnerability to faults. This lack of coordination can significantly impact the system’s reliability and overall performance.

#### Lack of evaluation and punishment mechanism

Existing systems often lack effective reputation mechanisms to evaluate and incentivize nodes. Without such mechanisms, the system may struggle to maintain high standards of performance and reliability, leaving it vulnerable to malicious or suboptimal nodes. Traditional systems also struggle with detecting and mitigating malicious nodes. Techniques such as random partial checking and trap messages are employed to detect faults but do not provide robust solutions for penalizing or preventing malicious activities. Random partial checking offers only probabilistic guarantees, while trap messages can identify the presence of a malicious node but not its specific source.

#### High computational costs

The computational costs associated with multi-signatures can be substantial, which poses challenges to system efficiency and scalability. Multi-signature schemes often require extensive cryptographic operations to sign and verify messages, leading to increased processing time and resource consumption. This can be particularly problematic in large-scale networks where frequent communication and numerous transactions are expected. High computational overhead can slow down the system and increase latency, impacting the user experience and system performance. To address this, improved multi-signature schemes with public key aggregation are needed to optimize performance. These advanced schemes aim to reduce the computational burden by aggregating multiple signatures into a single, compact form, thus lowering the overall resource usage and enhancing the system’s scalability while maintaining robust security standards.

#### Our contributions

This paper offers several key contributions to anonymous communications:

- To address the problem of insufficient effective node evaluation and incentive mechanisms within the system, we introduce a novel reputation-based mechanism in SRFACS. This mechanism dynamically updates the node groups based on their historical activity and reliability. By evaluating nodes through their past performance, the system not only helps in identifying and mitigating faulty or malicious nodes but also encourages all nodes to maintain high standards of performance and reliability.
- SRFACS enhances fault tolerance and manages single points of failure by incorporating an improved Asynchronous Byzantine Fault Tolerance (ABFT) protocol. We use this protocol to expand the traditional communication nodes into structured node groups and handles up to (*n* − 1)/3 faulty nodes within these groups, enhancing fault tolerance and maintaining continuous operation, especially in asynchronous environments. With ABFT, SRFACS ensures continuous and reliable operation even in the presence of numerous faulty nodes, thereby improving the overall robustness of anonymous communication systems.
- To efficiently manage inter-group communication, SRFACS utilizes leader nodes to coordinate and streamline communication processes. To mitigate the risks associated with leader failures, we have implemented an efficient leader change protocol that promptly replaces defective leaders, ensuring uninterrupted system performance. Additionally, to prevent erroneous leader actions from compromising the system, we have introduced an advanced multi-signature scheme. This approach secures communication and reduces computational overhead by requiring multiple signatures for verification processes. Through rigorous security analysis and component performance evaluation, we can confirm that SRFACS shows the potential of scalability and effectiveness in anonymous communication networks.

The rest of this paper is organized as follows. In the Building Blocks section, we detail the building blocks used in our SRFACS scheme, including cryptographic techniques and protocols. The SRFACS Preliminary section outlines the assumptions and foundational elements needed to ensure the system’s secure operation. In the SRFACS Design section, we present our approach to integrating various components, including the reputation mechanism, the ABFT protocol, and the multi-signature process. The Security Analysis section assesses the robustness and reliability of the system against various threats. The Performance Evaluation section provides a detailed analysis of SRFACS’s performance, focusing on tests of key components to demonstrate the effectiveness of our methods. Finally, the Conclusion section summarizes our findings and discusses potential future research directions.

## Building blocks

This section outlines the various components utilized in our work, detailing their individual functions and specific characteristics.

### Signature algorithm

#### MSP (Pairing-based multi-signature with public-key aggregation)

We adopt the MSP scheme proposed by [32], which is based on bilinear pairings. This scheme achieves signature aggregation and key aggregation and is resilient against rogue key attacks [33]. In order to better adapt to our system, we have made adjustments. As shown in Algorithm 1, the description is as follows:

**Algorithm 1** MSP Signature Scheme

1: **System Initialization:**

2: Generate security parameters and public parameters:

3:  params = (*q*, *G*_1_, *G*_2_, *G*_*t*_, *e*, *g*_1_, *g*_2_)

4:

5: **Key Generation:**

6: **for** each signer *i* **do**

7:  Generate private key:

8:   $msk_{i}\in Z_{q}$

9:  Compute public key:

10:   $mpk_{i}=g_{2}^{msk_{i}}$

11: **end for**

12:

13: **Key Aggregation:**

14: Aggregate public keys:

15:  $apk=\prod mpk_{i}^{a_{i}}$

16:

17: **Signature Generation:**

18: Map message *m* to a group element:

19:  *H*(*m*) ∈ *G*_1_

20: **for** each signer *i* **do**

21:  Generate partial signature:

22:   $s_{i}=H{\left(m\right)}_{0}^{a_{i}*msk_{i}}$

23: **end for**

24:

25: **Signature Aggregation:**

26: Aggregate partial signatures:

27:  *agg* = ∏*s*_*i*_

28:

29: **Signature Verification:**

30: Verify if:

31:  $e\left(agg,g_{2}^{-1}\right)\cdot e\left(H_{0}\left(m\right),apk\right)=1_{G_{t}}$

MSP.Init: Pg(λ) initializes the parameters par to establish a bilinear group (*q*, *G*_1_, *G*_2_, *G*_*t*_, *e*, *g*_1_, *g*_2_), where *G*_1_, *G*_2_, and *G*_*t*_ are groups of prime order *q*, and *g*_1_ and *g*_2_ are generators of *G*_1_ and *G*_2_ respectively. The function *e* denotes a valid non-degenerate bilinear mapping *e*: *G*1 × *G*_2_ → *G*_*t*_. Additionally, the hash functions *H*_0_ and *H*_1_ and the parameter par are output as follows:

$$\begin{array}{c} H_{0}:{\left\{0,1\right\}}^{*}\to G_{1},\;H_{1}:{\left\{0,1\right\}}^{*}\to Z_{q}\text{par}\leftarrow \left(q,G_{1},G_{2},G_{t},e,g_{1},g_{2}\right) \end{array}$$

MSP.KeyGen: After initialization, each node *i* generates a unique key pair according to the following formula: a private key *msk*_*i*_, a public key *mpk*_*i*_, by selecting a random number random, and then publishes the public key to the network, where *i* represents the Node ID.

$$\begin{array}{c} msk_{i}\overset{\;\text{random}\;}{\leftarrow }(Z_{q}),\;mpk_{i}\leftarrow g_{2}^{msk_{i}}\in G_{2} \end{array}$$

MSP.AggMpk(*mpkList*) → *apk*: Generate the aggregated public key *apk* according to the following formula, where *mpkList* is the set of public keys, *a*_*i*_ ← *H*1: (*mpk*_*i*_, {*mpk*_1_, …, *mpk*_*n*_}).

$$\begin{array}{c} apk\leftarrow \prod_{i=1}^{n}mpk_{i}^{a_{i}} \end{array}$$

MSP.Sig(*par*, *mpkList*, *msk*, *m*) → *s*_*i*_: Nodes use *par*, *mpkList*, *msk*, and *m* to generate a BLS signature for the current round message *m* and send it to the Leader.

$$\begin{array}{c} s_{i}\leftarrow H_{0}{\left(m\right)}^{a_{i}\cdot msk_{i}} \end{array}$$

MSP.Agg({*s*_*i*_}) → *agg*: The leader node generates the aggregated signature *agg* upon receiving the signatures {*s*_*i*_}.

$$\begin{array}{c} agg\leftarrow \prod_{j=1}^{n}s_{j} \end{array}$$

MSP.AggVf(par, apk, agg, m) → 1:Inputs par, apk, agg, m, output 1 if and only if the following equation holds true, indicating successful verification and valid signature:

$$\begin{array}{c} e\left(agg,g_{2}^{-1}\right)\cdot e\left(H_{0}\left(m\right),apk\right)=1_{G_{t}} \end{array}$$

### Asynchronous Byzantine Fault Tolerance (ABFT)

In Asynchronous Byzantine Fault Tolerance (ABFT) systems, various cryptographic techniques and protocols ensure secure consensus even in the presence of faulty nodes. Below, we introduce key components of ABFT, including Threshold Encryption, Byzantine Reliable Broadcast (RBC), and Asynchronous Binary Agreement (ABA).

#### Threshold encryption

Threshold Public-Key Encryption (TPKE) is a cryptographic primitive that enables any party to encrypt a message using a master public key [34, 35], requiring network nodes to collaboratively decrypt it. The interface of a threshold scheme includes:

- **TPKE.Setup**(1^λ^) → PK, SK_*i*_: Generates a public encryption key PK and secret keys for each party *SK*_*i*_.
- **TPKE.Enc**(PK, m) → C: Encrypts a message *m*.
- **TPKE.DecShare**(SK_*i*_, c) → *σ*_*i*_: Produces the *i*-th share of the decryption.
- **TPKE.Dec**(PK, C, i, *σ*_*i*_) → m: Combines decryption shares i, *σ*_*i*_ from at least *f* + 1 parties to recover the plaintext *m*.

#### Byzantine reliable broadcast (RBC)

In RBC, a designated sender (one of the replicas) disseminates a message to all other replicas. The protocol ensures:

- Agreement: If two correct replicas receive messages *m* and *m*′, then *m* = *m*′.
- Totality: If some correct replica delivers a message *m*, all correct replicas deliver *m*.
- Validity: When a correct sender broadcasts a message *m*, it will be received by all correct replicas.
- Integrity: Every correct replica delivers a message *m* from sender *p* at most once. If *p* is correct, then *m* was previously broadcast by *p*.

#### Asynchronous binary agreement (ABA)

ABA enables replicas to agree on a single bit value, exhibiting the following properties:

- Validity: If all correct replicas have the same input value *v*, correct replicas will deliver *v*.
- Agreement: If a correct replica delivers *v* and another correct replica delivers *v*′, then *v* = *v*′.
- Termination: All correct replicas eventually deliver a binary value with probability 1.

**Algorithm 2** Leader Change Protocol

1: **procedure ** Leader Change Protocol

2:  **Monitoring Phase:**

3:  **while** True **do**

4:   Leader node *k* sends heartbeat messages to other nodes

5:   **if** node *i* does not receive heartbeat from *k* within timeout **then**

6:    Initiate leader change

7:    **break**

8:   **end if**

9:  **end while**

10:

11:  **Broadcasting Phase:**

12:  Node *i* broadcasts 〈Request, *j*, *k*, *i*〉 to all nodes

13:  Select node *j* with the highest RS score as the next candidate leader

14:

15:  **Update Phase:**

16:  **if** node *j* receives *f* + 1 request messages **then**

17:   Consensus on leader change is reached

18:   Node *j* broadcasts 〈Reply, *j*, *k*, *C*〉 to all nodes

19:   All nodes update their leader node information to *j*

20:   All nodes inform the management center (MC)

21:   **if** MC receives 2*f* + 1 change messages **then**

22:    MC updates the node information list

23:    MC sets the RS of node *k* to zero

24:    Node *j* starts its operation as the new leader

25:   **end if**

26:  **end if**

27: **end procedure**

### Leader change protocol

AsynGroup relies on a properly functioning leader node. To prevent leader node failure, we have designed the Leader change protocol. Since the leader node is the highest-quality node in the system, we do not consider malicious behavior from it. Specific description is shown in Algorithm 2.

#### Monitoring phase

The leader node *k* periodically sends heartbeat messages to other nodes within AsynGroup, declaring itself as the Leader node, while other non-Leader nodes also wait to receive heartbeat messages. Certain circumstances may prevent these messages from being received, indicating a potential failure of the leader node *k*. If node *i* does not receive heartbeat messages from the leader node *k* within a certain period, it is assumed that the leader node has failed, and a request for a leader node change is initiated.

#### Broadcasting phase

Node *i* broadcasts a message requesting node change <*Request*, *j*, *k*, *i*>to all replicas within AsynGroup, where *j* is the next candidate (with the highest RS score) node ID. If there are nodes with the same score, the one with the smaller Node ID is given priority.

#### Update phase

Upon receiving *f* + 1 request node change messages, the new leader node *j* considers consensus reached for node change. The new leader node *j* broadcasts a reply message <*Reply*, *j*, *k*, *C*>to notify all nodes of the leader node change. This message includes the new Leader node information *C*. All nodes within AsynGroup update their local leader node information lists accordingly.

All nodes send leader node change information <*Change*, *j*, *k*, *i*>to MC. After receiving 2*f* + 1 change messages, MC updates the node information list, sets the RS of the failed leader node *k* to zero, and the new leader node begins operation. Communication terminates during protocol execution, and after the protocol ends, MR will initiate a new communication request.

### Reputation incentive mechanism

To achieve a reasonable configuration of AsynGroup, we designed a node reputation incentive mechanism. We select nodes with high reputation values from the reputation list as candidate nodes, considering them to be high-quality nodes. Our core idea involves maintaining a node information list, which records the reputation value of each node. We assign an integer identifier “ID” to each node. All replicas maintain a node information list obtained from MC, which records the ID, IP, status, and RS of all nodes, as shown in Table 2.

**Table 2:** Node information list.

ID	IP	Status	RS
1	192.168.0.1	Leader	1
2	192.168.0.2	Common	1
3	192.168.0.3	Common	1
4	192.168.0.4	Common	1
…	…	…	…

Dynamic updating of nodes participating in anonymous communication systems can enhance system security. However, there are no specific standards to determine the priority of node selection. Therefore, in our protocol, we have designed a reputation incentive mechanism to address this issue. We define a reputation score (RS) as a metric to measure the credibility of nodes [36].

#### Reputation update

In the protocol, each node is required to maintain a local node information list obtained from MC. The initial RS of all nodes in MC is set to 1. At the beginning of each epoch, we calculate the RS of network nodes based on events from the previous epoch. The process is detailed as follows:

1. Node Evaluation: Our reputation score (RS) is calculated based on events throughout the entire epoch. The reputation score (RS) is used as the criterion for selecting nodes as candidates for the next epoch’s AsynGroup. In our reputation model, we have identified three important parameters for this evaluation along with their respective weight coefficients:

1)Participation Degree: During anonymous communication, we consider whether nodes actively participate in the AsynGroup’s intra-group asynchronous ABFT consensus during the recent anonymous communication rounds [37]. Participation is partially defined as follows:

$$\begin{array}{c} E=\{\begin{array}{cc} SF, & \text{Execute}\;\text{ABFT}\;\text{successful}\\ UF, & \text{Otherwise} \end{array} \end{array}$$ (1)

Our parameter *S*_*p*_ is defined as Eq (2):

$$\begin{array}{c} S_{p}\left(i\right)=\frac{SF}{\left(SF+UF\right)} \end{array}$$ (2)

2) Credit: If a node can correctly complete the MSP signature algorithm process, its credibility will be higher. We define the terms as follows:

#### Complete signature correctly

When a node’s aggregated signature passes the MSP. AggVf verification, we consider the node to have completed the signing process correctly.

*C*_*sc*_ represents the number of correct completions by the node in the recent rounds. *C*_*se*_ represents the number of error completions by the node. *T*_*total*_ represents the total number of communications in the recent rounds, where *γ*_1_ and *γ*_2_ are weight parameters. *γ*_2_ is much larger than *γ*_1_. The parameters are defined as Eq (3):

$$\begin{array}{c} S_{c}\left(i\right)=\gamma_{1}\times \frac{C_{sc}}{T_{total}}-\gamma_{2}\times \frac{C_{se}}{T_{total}} \end{array}$$ (3)

3) Bandwidth: We consider the average network bandwidth performance of nodes in recent rounds (Bi) and set the threshold level Bt to 500 Mbps. The parameter is defined as Eq (4):

$$\begin{array}{c} S_{t}\left(B_{i}\right)=\frac{1}{1+|B_{t}-B_{i}|} \end{array}$$ (4)

2. Update: Our reputation updates are based on events in each round, to better track node behavior at different stages, mitigate the long-term impact of early node behavior on reputation, and more accurately reflect its current reputation status, we assume that the current round in which AsynGroup nodes start participating in anonymous communication is denoted as *v*. After passing through *k* rounds, it reaches *v*1, and after passing through another *k* rounds, it reaches *v*′. At this point, one epoch is completed, updating all AsynGroup nodes, and MC reconfiguring AsynGroup. We divide the evaluation into two stages, that is, reputation is evaluated every *k* rounds. For example, the evaluation of the first *k* rounds of *v*1 is as Eq (5):

$$\begin{array}{c} RS{\left(i\right)}_{v1}=\beta_{1}\times S_{p}{\left(i\right)}_{v1}+\beta_{2}\times S_{c}{\left(i\right)}_{v1}+\beta_{3}\times S_{t}{\left(B_{i}\right)}_{v1} \end{array}$$ (5)

Following the combination of reputation evaluations in two stages, our update formula is as Eq (6):

$$\begin{array}{c} RS\left(i\right)=\left[S{\left(i\right)}_{v^{\prime }}\right]\left(1-\chi \right)+S{\left(i\right)}_{v1}\times \chi \end{array}$$ (6)

where *χ* is the forgetting factor:

$$\begin{array}{c} \chi =\frac{1}{1+e^{-(v^{\prime }-v)}}\in \left(0,1\right) \end{array}$$ (7)

After completing an entire Epoch, the system collects the above-mentioned metric data for nodes. Based on the algorithm, it calculates the RS of each node and stores it in the system.

AsynGroup Update: After evaluating all nodes, MC sorts the nodes in the node information list from high to low based on RS (if RS is the same, nodes with smaller IDs are placed in front). The top 36 nodes in the node information list are selected as candidate high-scoring nodes for the next Epoch’s AsynGroup, with the top four nodes being leaders of each group. MC will add the selected new high-scoring nodes to the system in order. Each node updates its local list based on MC’s information. After the update, the system reconfigures the network settings of the nodes to ensure that they can connect to the system properly.

Through the above implementation steps, the system can dynamically configure nodes participating in anonymous communication better, selecting high-quality nodes based on their reputation scores RS to improve the security and performance stability of the system.

## SRFACS preliminary

### Adversary model

We assume that all nodes use authenticated channels for communication. This ensures that while an adversary can eavesdrop, forward, and delete messages between nodes, they cannot modify, replay, or inject new messages without detection. For communications not involving nodes, the adversary has the ability to eavesdrop, modify, or inject messages at any point in the network.

We assume at least two users will stay honest.Similarly, nodes can be compromised, but at least one SRFACS GRoup must remain honest to ensure the system’s security. Compromised nodes are considered malicious but cautious, avoiding detection while violating the protocol.

The adversary seeks to break the anonymity of the initiator or link inputs and outputs. As with other anonymous communication, we do not consider adversaries focused solely on launching DoS attacks.

We propose a deployment model utilizing dedicated, trusted data centers as nodes, potentially selected through competitive processes. These centers have strong incentives not to be expelled. This contrasts with some anonymous communication where nodes can enter and leave the network with minimal cost.

In our deployment model, it is sufficient to detect cheating nodes with a high probability eventually. In more flexible deployment models, nodes must more rigorously prove correct computation before the output is revealed. SRFACS ensures that the exit node commits to its output before it can read the system output, protecting against certain adaptive attacks.

### Security goals

SRFACS aims to achieve two primary security properties:

Anonymity: A protocol guarantees anonymity if the adversary is unable to link any input message to its corresponding output message with a probability significantly better than random guessing, even when the adversary compromises all but two users and all but one node.

Integrity: A protocol ensures integrity if, at the end of each execution involving *β* honest users, it satisfies one of the following conditions: Messages sent by *β* honest users reach their intended recipients without any alteration. The probability of detecting a malicious node is high, ensuring that no honest party is mistakenly flagged as malicious.

## SRFACS design

### Details of SRFACS

**Fig 1:**
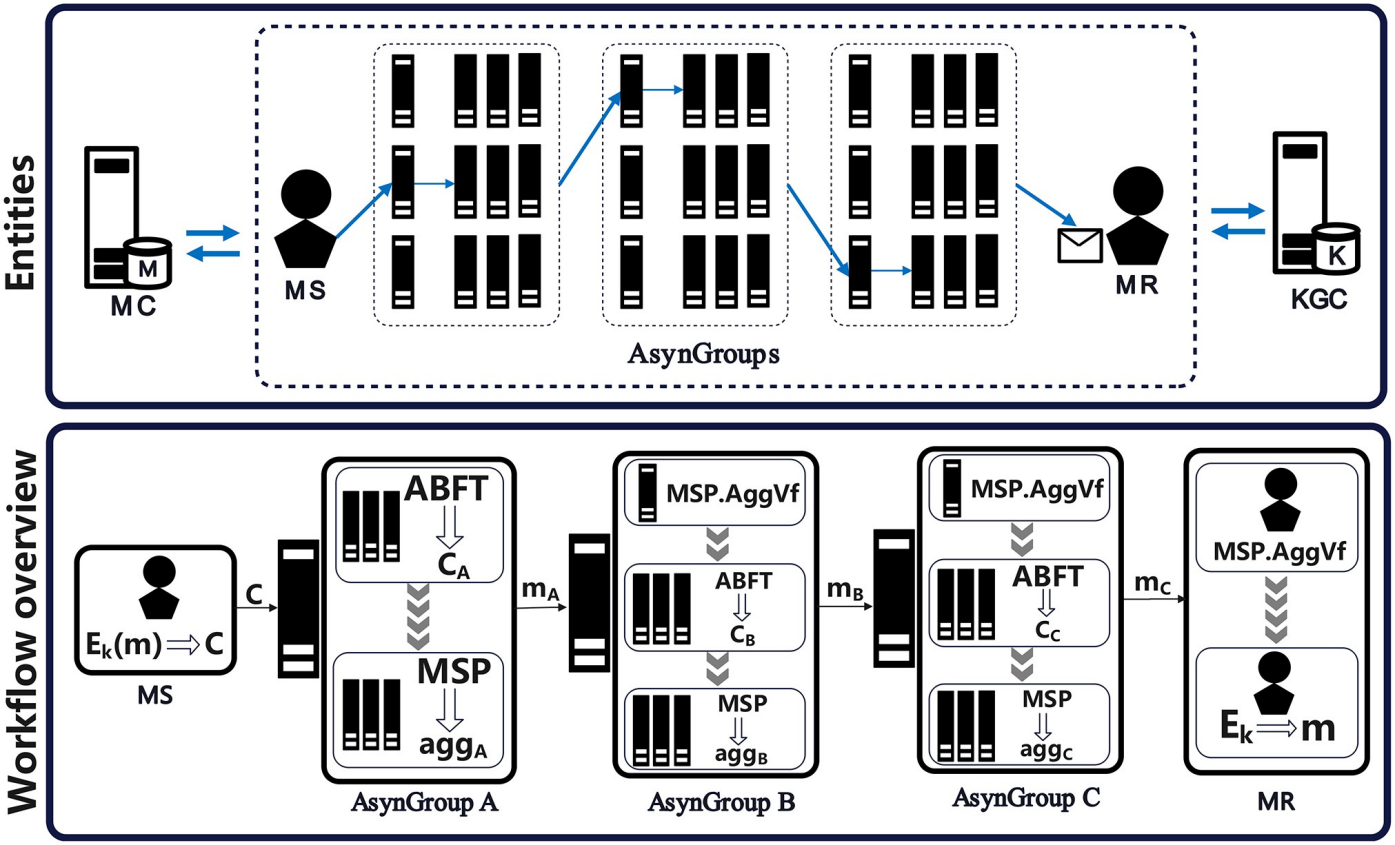
System overview of SRFACS. The messages *m*_*A*_ and *m*_*B*_ are defined as *m*_*A*_ = 〈*C*_*A*_, *agg*_*A*_, *apk*_*A*_〉 and *m*_*B*_ = 〈*C*_*B*_, *agg*_*B*_, *apk*_*B*_〉.

As shown in Fig 1, our system includes the following entities.

1. **Management Center (MC):** Serving as the fully trusted entity within the management system, the MC monitors and records the behavior of each node. It computes node reputation, configures AsynGroup, and synchronizes node address information.

2. **Key Generation Center (KGC):** Functioning as a trusted entity, the KGC generates public parameters and collaboratively produces user partial keys.

3. **Message Sender (MS):** The MS encrypts and encapsulates the message for transmission.

4. **Message Receiver (MR):** Acting as the recipient of information, the MR unpacks messages to retrieve plaintext.

5. **AsynGroups:** Anonymous communication propagation node group is composed of 36 high reputation nodes. We divide AsynGroups into three groups, namely AsynGroups A, AsynGroups B and AsynGroups C. Each AsynGroup contains three AsynGroups, and each AsynGroup is composed of three common nodes and a master node. MS will select one AsynGroup from each AsynGroup. These three AsynGroups are recorded as AsynGroup A, AsynGroup B, and AsynGroup C.

#### Workflow of SRFACS

As shown in Fig 1, our system includes the following steps.

**Algorithm 3** System Setup

1: **Initialization:**

2:  *Rounds*← Partition time into discrete units

3:  *Epochs* ← 2*k*

4:

5: **Key Generation:**

6:  Register nodes (MS, MR, AsynGroup) at key generation center

7:  Generate keys using:

8:   MSP.Init, MSP.KeyGen, TPKE.Setup

9:  Distribute generated keys to nodes

10:

11: **AsynGroup Configuration:**

12:  Sort nodes by RS at Management Center (MC)

13:  Select top 36 nodes as AsynGroup candidates

14:   Designate top 9 nodes as primary nodes

15:

16: **Node Integration:**

17:  Integrate high-scoring nodes into the system

18:  Update local node lists and network configurations

1. **System setup:** As shown in Algorithm 3, we partition time into discrete units known as Round and segment the operation of the entire system into continuous Epoch, with each Epoch comprising 2k Rounds. Each Round embodies a comprehensive anonymous communication process, while each Epoch signifies a full cycle of the system, We introduce the Symbols throughout the SRFACS in Table 3.

**Table 3:** Description of symbols.

Symbol	Symbol Definition
λ	security parameter
mpk/msk	MSP key pairs
PK/SK	TPKE key pairs
A_1_/A_2_/A_3_/A_r_	primary nodes and MR address
m/c	message m and ciphertext of m
*E*_*k*_	communication symmetric key
A_Leader_/B_Leader_/C_Leader_	A/B/C group’s leader node
*σ*_*i*_	decryption share

At the onset of each Epoch, we undertake the updating of AsynGroup to enhance the capability of system nodes in fulfilling anonymous communication tasks.

During AsynGroup node configuration, the Management Center (MC) orchestrates the sorting of nodes based on their reputation score (RS). The RS serves as a reflection of the node’s credibility and reliability within the system. Leveraging these RS metrics, MC selects the top 36 nodes from the node information list as candidate AsynGroup nodes. Among these 36 nodes, the top nine are designated as candidate primary nodes. These master nodes assume the responsibility of coordinating and overseeing their respective node combination to ensure normal communication among nodes.

**Algorithm 4** Send Phase

1: **Send Phase:**

2: **Initialization:**

3:  *m*← message to be transmitted

4:  *pk*_*A*_, *pk*_*B*_, *pk*_*C*_← public keys of current AsynGroup

5:  *A*_*Leader*_← Leader node of AsynGroup A

6:  *E*_*k*_← symmetric key for encryption

7:  *A*_1_← Master node address

8:  *C*← ciphertext

9:

10: **Message Encryption:**

11:  *E*_*k*_(*m*)← Encrypt message *m* using symmetric key *E*_*k*_

12:  Encrypt *E*_*k*_(*m*) and address using threshold public key encryption with *pk*_*C*_, *pk*_*B*_, *pk*_*A*_

13:  *C* ← *pk*_*A*_(*pk*_*B*_(*pk*_*C*_(*E*_*k*_(*m*), *A*_*r*_), *A*_3_), *A*_2_), *A*_*r*_

14:

15: **Message Transmission:**

16:  Dispatch ciphertext *C* to *A*_*Leader*_ based on address *A*_1_

After determining the members and leader nodes of AsynGroup, the identified high-scoring nodes are integrated into the system. Each node then updates its local list by the latest node information list of the system. Upon completion of the update process, MC reconfigures the network settings of nodes to facilitate accurate system connection and sustained communication among nodes.

2. **Key Generation:** Message Sender (MS), Message Receiver (MR), and every node in AsynGroup are registered within the key generation center. The key generation center employs the MSP.Init, MSP.KeyGen, and TPKE.Set up algorithms to generate global parameters and corresponding keys for users and nodes, subsequently distributing keys to the corresponding entities.

3. **Send phase:** As shown in Algorithm 4, MS encrypts the transmitted message *m*. Initially, MS acquires the master node address and the public keys *pk*_*C*_, *pk*_*B*_, and *pk*_*A*_ of the current AsynGroup from MC. Subsequently, MS utilizes the symmetric key *E*_*k*_ to encrypt the message *m*. Following this, MS employs the threshold encryption algorithm TPKE Enc along with the public keys *pk*_*C*_, *pk*_*B*_, and *pk*_*A*_ to encrypt *E*_*k*_(*m*) and the address. The resultant encrypted ciphertext *C* is then dispatched to the *A*_*Leader*_ of AsynGroup A based on the address *A*_1_. The ciphertext *C* is structured as follows:

$$\begin{array}{c} C=\langle pk_{A}\langle pk_{B}\langle pk_{C}\langle E_{k}\left(m\right)∥\left(A_{r}\right)\rangle ∥\left(A_{3}\right)\rangle ∥\left(A_{2}\right)\rangle \rangle \end{array}$$

**Algorithm 5** MSP Signature Process

1: **After achieving consensus through ABFT protocol:**

2:

3: **Message Signing:**

4:  Each consensus node within group A signs the message *C*_*A*_

5:  using MSP.Sig to obtain signature *S*_*i*_

6:

7: **Broadcast Signed Message:**

8:  Nodes broadcast signed message 〈*S*_*i*_, *i*〉 to *A*_*Leader*_

9:

10: **Key Aggregation:**

11:  *A*_*Leader*_ aggregates public keys *apk*_*A*_ of each node

12:  using MSP.AggMpk

13:

14: **Signature Aggregation:**

15:  *A*_*Leader*_ runs MSP.Agg to obtain aggregated signature *agg*_*A*_

16:

17: **Message Broadcast:**

18:  Broadcast message 〈*C*_*A*_, *agg*_*A*_, *apk*_*A*_〉

19:  using Byzantine Reliable Broadcast to *B*_*Leader*_ within group B

4. **Transmission phase:** As shown in Algorithm 5 and Algorithm 6, this stage primarily consists of two components: 1. ABFT protocol, 2.MSP algorithm. We illustrate the propagation process of AsynGroup A as an example.

1) **ABFT protocol:** When the *A*_*Leader*_ of AsynGroup A receives the ciphertext *C* from MS, the nodes start executing the asynchronous BFT protocol, which includes Byzantine Reliable Broadcast (RBC) and Asynchronous Binary Agreement (ABA). Upon the conclusion of the asynchronous BFT protocol, each correct node within group A maintains the same message:

$$\begin{array}{c} \left\langle pk_{A}\langle pk_{B}\langle pk_{C}\langle E_{k}\left(m\right)∥\left(A_{r}\right)\rangle ∥\left(A_{3}\right)\rangle ∥\left(A_{2}\right)\rangle \right\rangle \end{array}$$

**Fig 2:**
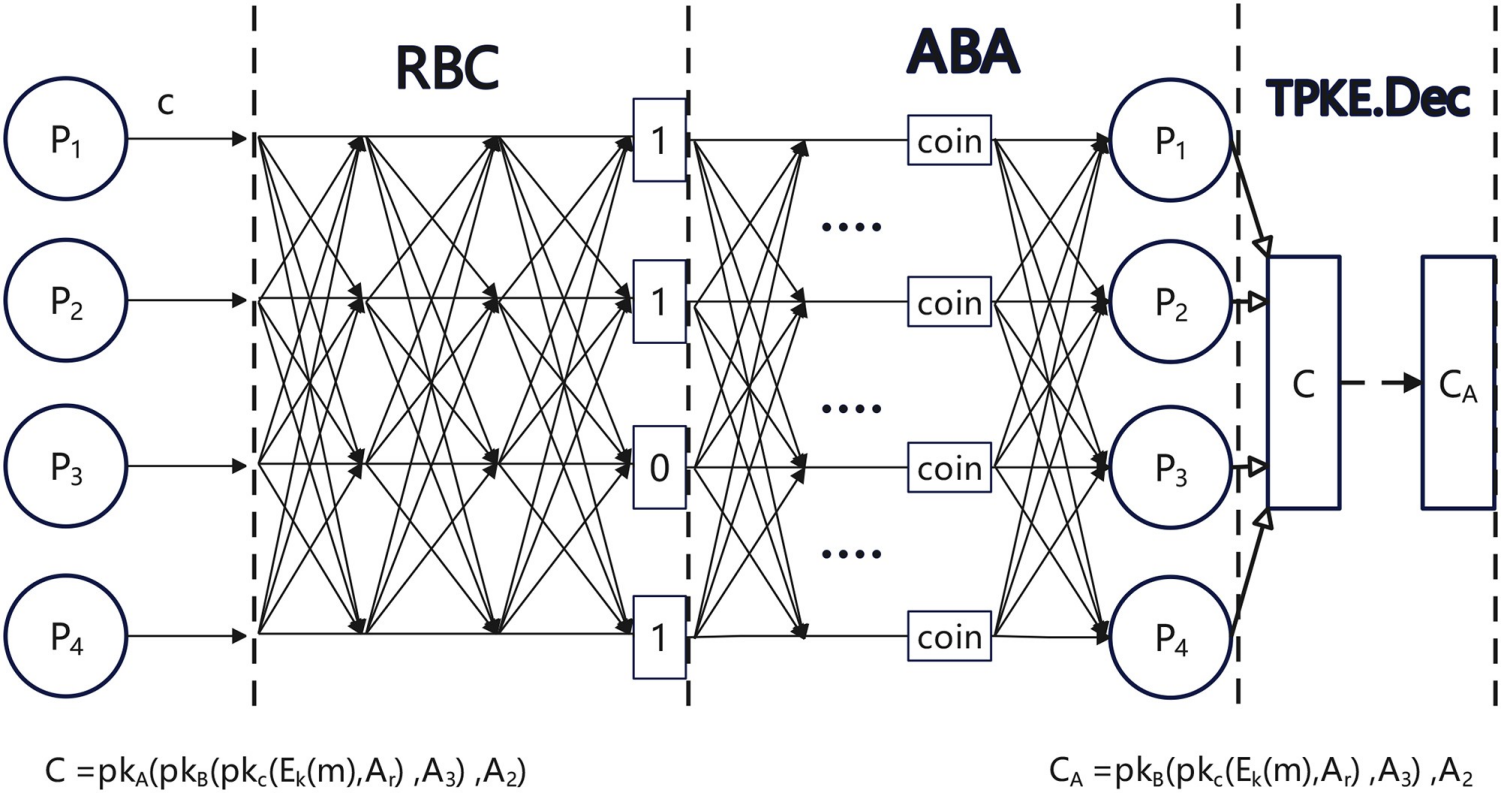
The message flow in AsynGroup A.

Each correct node can generate a decryption share *σ*_*i*_ using the threshold encryption scheme *TPKE*.*DecShare*. Subsequently, each correct node combines the decryption shares using *TPKE*.*Dec* to produce plaintext with threshold encryption. The plaintext is shown below. The message flow is shown in Fig 2.

$$\begin{array}{c} C_{A}=\langle pk_{B}\langle pk_{C}\langle E_{k}\left(m\right)∥\left(A_{r}\right)\rangle ∥\left(A_{3}\right)\rangle ∥\left(A_{2}\right)\rangle \end{array}$$

**Algorithm 6** Transmission and Receive Phase

1: **Transmission Phase:**

2: **Input:** Ciphertext *C* from MS

3: **Output:** Decrypted message *C*_*A*_

4:

5: **ABFT Protocol:**

6: **Byzantine Reliable Broadcast (RBC):**

7:  **SendToAll**(*C*)    ▷ Broadcast ciphertext to all nodes in AsynGroup A

8:  **WaitUntilAllReceived**(*C*)    ▷Ensure each correct node receives *C* from *A*_*Leader*_

9:

10: **Asynchronous Binary Agreement (ABA):**

11:  *C*_*A*_← **ABA**(*C*)    ▷Nodes execute ABA protocol to agree on the message *C*_*A*_

12:

13: **Decryption:**

14:  Each correct node generates decryption share *σ*_*i*_ using TPKE.DecShare

15:  Combine decryption shares using TPKE.Dec to produce plaintext with threshold encryption

16:  Plaintext: *C*_*A*_ = *pk*_*B*_(*pk*_*C*_(*E*_*k*_(*m*), *A*_*r*_), *A*_3_), *A*_2_

17:

18: **Receive Phase:**

19: **Upon receiving message *C*_*C*_:**

20:

21: **Signature Verification:**

22:  Verify the signature of *C*_*C*_

23:

24: **Decryption:**

25:  Decrypt the ciphertext *E*_*k*_(*m*) using the symmetric key *E*_*k*_ to retrieve the message *m*

26:

27: **Note:**

28:  The system design ensures that adversaries can listen passively but cannot discern specific message exchanges.

2) **MSP algorithm:** After the nodes within group A achieve consensus through the asynchronous BFT protocol, the consensus nodes within the group utilize MSP.Sig to sign the message *C*_*A*_ and obtain the corresponding signature *S*_*i*_. Nodes then broadcast the signed message <*S*_*i*_, *i*>to the leader node *A*_*Leader*_. Initially, *A*_*Leader*_ aggregates the public keys *apk*_*A*_ of each node using MSP.AggMpk and subsequently runs MSP.Agg to obtain the aggregated signature *agg*_*A*_. Following this, utilizing the address *A*_2_, the message <*C*_*A*_, *agg*_*A*_, *apk*_*A*_>is broadcasted using Byzantine Reliable Broadcast to the leader node *B*_*Leader*_ within group B.

As shown in Fig 1. After the communication within AsynGroup A as described above, when the leader node BLeader of AsynGroup B receives <*C*_*A*_, *agg*_*A*_, *apk*>from *A*_*Leader*_, it verifies the aggregated signature using MSP.AggVery. Upon successful verification, nodes in AsynGroup B execute the same ABFT protocol operations as AsynGroup A. Subsequently, each correct node within the group maintains the same plaintext: *C*_*B*_ = *pk*_*C*_(*E*_*k*_(*m*), *A*_*r*_), *A*_3_. Finally, the same MSP signature steps as AsynGroup A are executed to obtain the aggregated signature *agg*_*B*_. Then broadcast the message <*C*_*B*_, *agg*_*B*_, *apk*_*B*_>to the leader node *C*_*Leader*_ within group C. Similarly, AsynGroup C can verify and decrypt the message, obtaining *C*_*C*_ = *E*_*k*_(*m*), *A*_*r*_. Subsequently, it undergoes aggregation signature. As the message contains the address of MR, the leader node *C*_*Leader*_ within group C sends <*C*_*C*_, *agg*_*C*_, *apk*_*C*_>to MR. At this point, the message propagation phase concludes.

5. **Receive phase:** As shown in Algorithm 6, upon receiving message *C*_*C*_, MR first verifies the signature and then decrypts the ciphertext *E*_*k*_(*m*) using the symmetric key *E*_*k*_ to retrieve the message *m* sent by MS.This is the basic design of our system. The adversary can passively listen to the channel but he cannot tell this message from specific senders to the designated receivers.

## Security analysis

To assess the anonymity of the SRFACS protocol, we define an anonymity property through a game involving an adversary *A* and a challenger *C*. In this game, *A* is modeled as a probabilistic polynomial-time (PPT) Turing machine. The process starts with *C* executing the protocol and sharing the results with *A*. We assume a fixed number of *β* users, each sending a message in every round. Before the protocol runs, the adversary *A* can compromise all but two users and all but one node. The challenger *C* represents the honest nodes and users, while *A* controls the compromised entities. Although *A* can eavesdrop on the communications between nodes, it cannot alter them. Therefore, *C* provides *A* with copies of all communications between honest parties, including their sources and destinations. The output from this game for the adversary is denoted as *h*_*A*|*C*_.

The steps of the anonymity game are outlined as follows:

Setup Phase: The challenger *C* initializes all honest nodes and shares all public information with the adversary *A*.

Query Phase: For each query made by *A*, *C* receives input messages in the form of (sender, message) pairs for all *β* slots from *A*, and then executes the SRFACS protocol with those inputs.

Challenge Phase: The adversary *A* selects two honest users, *S*_0_ and *S*_1_, along with two distinct messages, $m_{0}^{\prime }$ and $m_{1}^{\prime }$. Additionally, *A* chooses messages for all other honest users, ensuring these messages are different from $m_{0}^{\prime }$ and $m_{1}^{\prime }$. This challenge is sent to *C*. The challenger *C* then flips a uniform random coin to obtain a bit *b*, assigning $m_{0}=m_{b}^{\prime }$ and $m_{1}=m_{1-b}^{\prime }$ to users *S*_0_ and *S*_1_, respectively. *C* runs the protocol using these input messages and provides the resulting output message set to *A*.

Query Phase (Post-Challenge): Following the challenge run, for each new query made by *A*, *C* takes the message inputs provided by *A* and re-runs the SRFACS protocol.

Output Phase: Finally, the adversary outputs *b*′ as its guess for *b*.

Proof: To prove the security of SRFACS, we reduce it to the security of the encryption system *E*. We utilize a modified version of the standard encryption game for defining the security of *E*. For a given challenge message pair (*m*_0_, *m*_1_), the encryption system’s challenger *C*_*E*_ returns a pair of ciphertexts (*E*_*pk*_(*m*_*b*_), *E*_*pk*_(*m*_1−*b*_)) instead of a single ciphertext *E*_*pk*_(*m*_*b*_).

Assuming without loss of generality that the adversary *A* can compromise up to *β* − 2 users and *n* − 1 nodes, we designate *S*_0_ and *S*_1_ as the two honest users and *i* as the sole honest node.

During the setup phase, the challenger *C* initiates the Chosen Plaintext Attack (CPA) game with the encryption challenger *C*_*E*_, ensuring the public key used in SRFACS matches that in the CPA-security game. In the query phase, *C* simulates the honest nodes, including their decryption shares. This simulation is feasible because *C* manages and verifies the hashing (random) oracle queries made by the adversary regarding messages and permutations, and can reveal the committed decryption shares (derived from a perfectly hiding scheme) using values it has selected.

In the challenge phase, the challenger *C* assigns the messages $m_{0}=m_{0\hat{b}}$ and $m_{1}=m_{0(1-\hat{b})}$, where $\hat{b}$ is a random bit. Additionally, *C* selects values *s*_*i*,0_ and *s*_*i*,1_ for the slots of *S*_0_ and *S*_1_ at honest node *i* such that the ratio *m*_0_/*m*_1_ = *s*_*i*,0_/*s*_*i*,1_. *C* then engages in the challenge phase with *C*_*E*_, using *s*_*i*,0_ and *s*_*i*,1_ as challenge messages. *C*_*E*_ responds with a ciphertext pair (*E*_*pk*_(*s*_*i*,*b*_), *E*_*pk*_(*s*_*i*,1−*b*_)). The anonymity-game challenger *C* incorporates this response pair into the computing phase for node *i* and slots *S*_0_ and *S*_1_, effectively embedding the CPA-security decryption challenge into an unknown permutation within the SRFACS anonymity game.

In the real-time operational phase of SRFACS, the challenger *C* follows the standard protocol, except at the mixing step for node *i*, where it uses *s*_*i*,1_ and *s*_*i*,0_ for slots *S*_0_ and *S*_1_ such that *m*_0_*s*_*i*,1_ = *m*_1_*s*_*i*,0_. This allows *C* to reveal any of the two honest real-time messages as either *m*_0_ or *m*_1_.

In the post-processing phase, the challenger *C* knows all permutations except for those corresponding to *m*_0_ and *m*_1_ (i.e., $m_{0\hat{b}}$ and $m_{0(1-\hat{b})}$). Consequently, *C* can reveal the decryption shares for all other messages. Since *m*_0_*s*_*i*,1_ = *m*_1_*s*_*i*,0_, *C* can open *m*_0_ and *m*_1_ in any sequence.

At the conclusion of the anonymity game, if the adversary *A* correctly guesses the bit $\hat{b}$, then the challenger *C* knows its selection of *s*_*i*,1_ and *s*_*i*,0_ for slots *S*_0_ and *S*_1_ in the real-time phase matched the challenge ciphertexts from the computing phase. In this case, *C* will output *b* = 1 to the encryption game challenger *C*_*E*_. Conversely, if *A*’s guess $\hat{b}$ is incorrect, *C* will output 0 to *C*_*E*_.

## Performance evaluation

### Signature process experiment

We assess the performance of the multi-signature component (MSP) in our proposed anonymous communication system, SRFACS. The signature process was evaluated on a single computer with the following specifications: an Intel(R) Core(TM) i5-13490F CPU, 32GB of RAM, running on Windows 10. We measured the performance by tracking the time taken for various stages, including key generation, signature generation, signature aggregation, and verification of the aggregated signature. The experiments were conducted using four different elliptic curve parameters: SS512, MNT159, MNT224, and BN254. Our experiment utilized the Charm-Crypto library [38]. Additionally, the number of nodes was varied to observe the scalability of the algorithm, with tests performed for 4, 8, 12, and 16 nodes.

#### Experimental results and analysis

**Fig 3:**
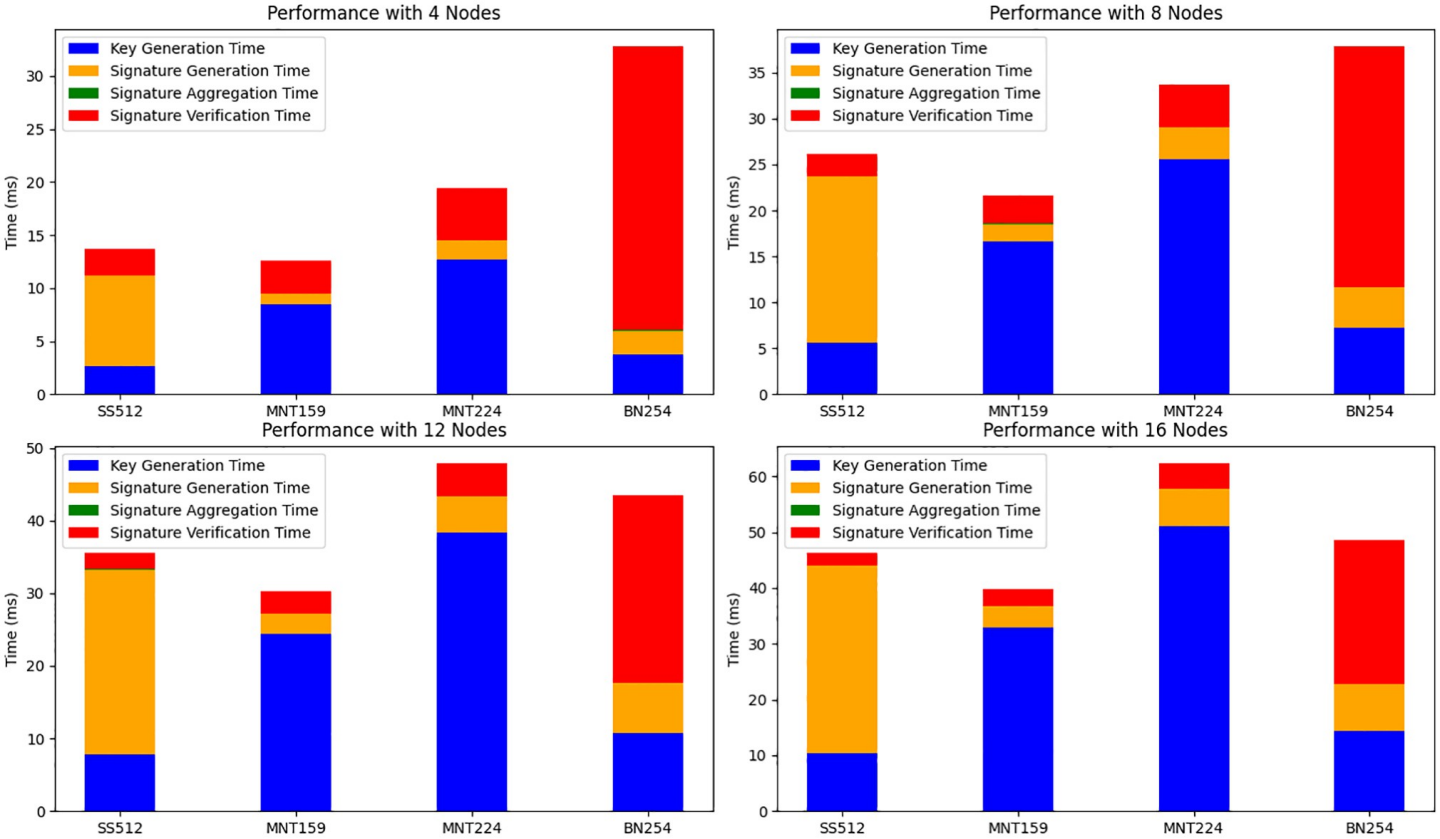
Time breakdown for each step of the MSP algorithm across different elliptic curves and varying numbers of nodes: 4, 8, 12, and 16.

Fig 3 presents the total time taken for the MSP algorithm across different elliptic curves, broken down into the key generation, signature generation, signature aggregation, and signature verification stages. The results demonstrate distinct performance differences between the curves and node sizes. The MNT159 curve consistently exhibited the lowest total time across all node configurations, primarily due to its fast key generation and signature verification times. SS512, while slightly slower, provided a balanced performance across all stages, making it a good choice for applications with moderate time constraints.

In contrast, BN254 showed the highest total time across all stages, particularly due to the high cost of signature verification, which was the most time-consuming step. This curve displayed a noticeable increase in total time as the number of nodes increased, indicating less scalability compared to the other curves. MNT224 also had a higher overall time, though it performed better than BN254 in key generation and signature aggregation.

The analysis highlights that our choice of elliptic curves and MSP method effectively reduces computational overhead, particularly for key generation and verification, which are crucial for real-time applications. MNT159 and SS512 stand out as the most efficient choices, especially as the number of nodes increases, suggesting that these curves are well-suited for applications requiring scalability. Furthermore, the total time remains manageable even with 16 nodes, demonstrating that the system can support a growing network size without significant performance degradation.

### Reputation component test experiment

Our experiment is based on the methodology presented in [39]. This method is used to simulate the running process of the individual Reputation component, calculate its time cost, and use this data as a reference.

#### Experimental results and analysis

**Fig 4:**
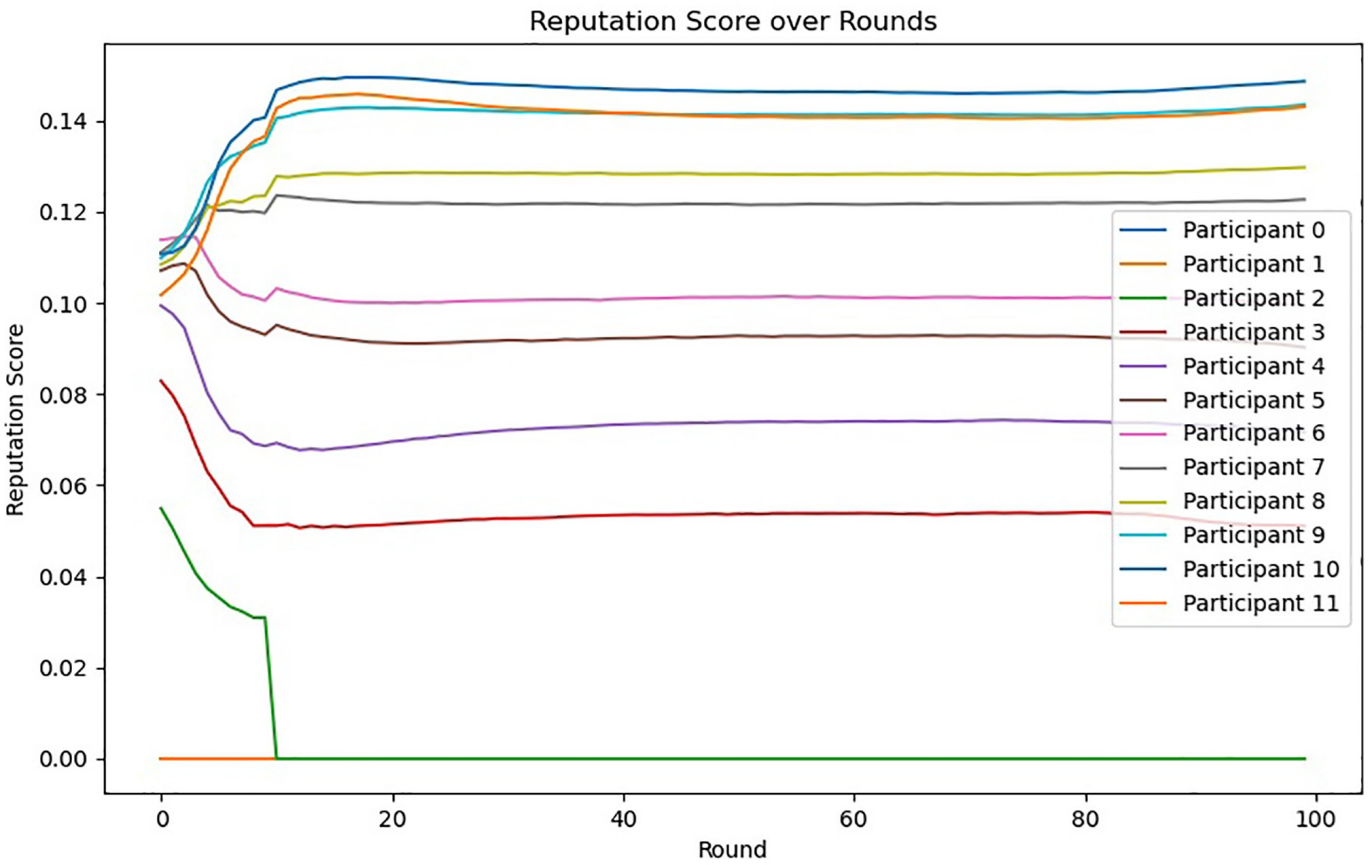
Reputation scores per round for all nodes.

**Fig 5:**
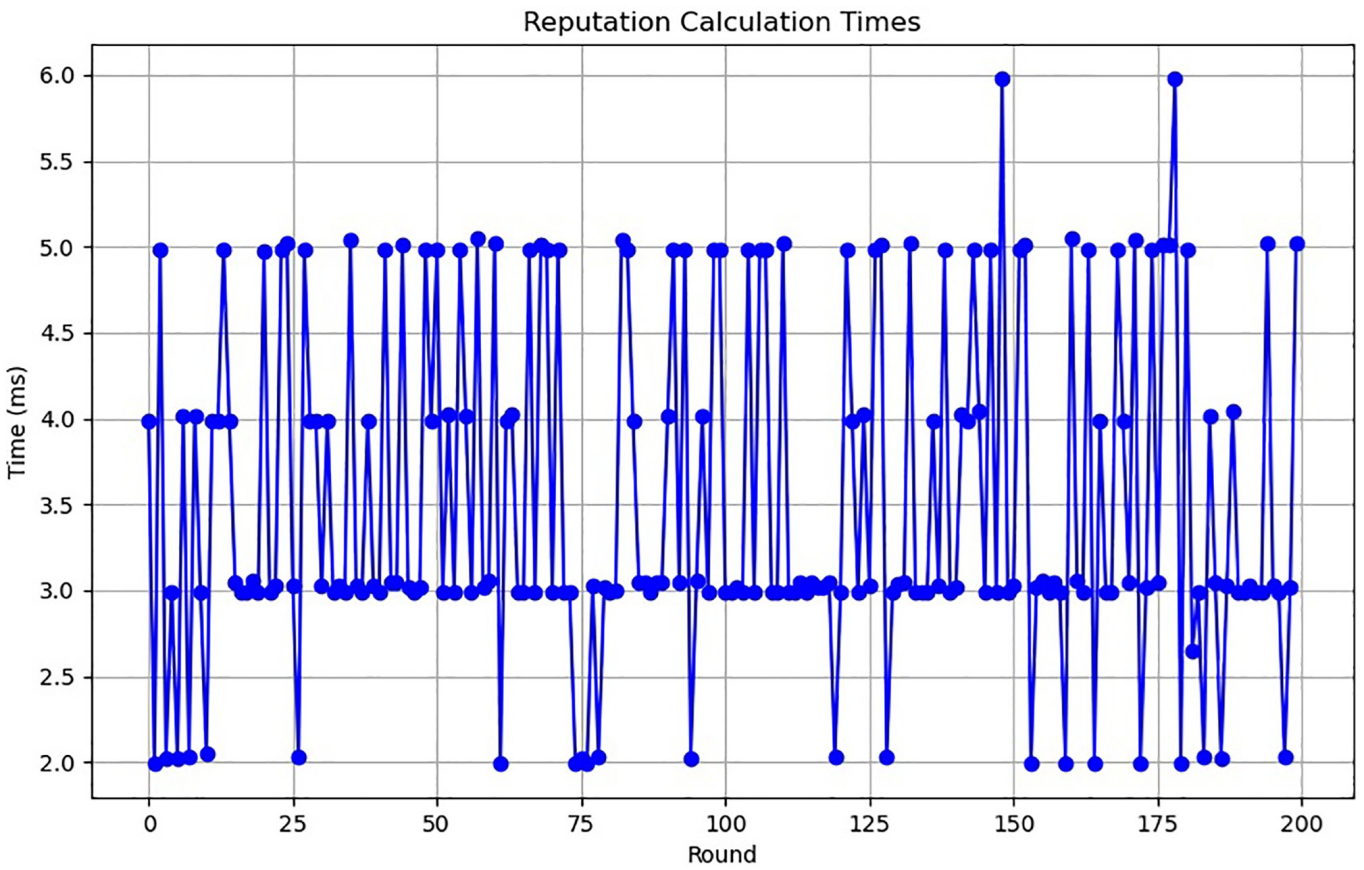
Reputation calculation time (in milliseconds) per round.

The results of the reputation mechanism testing are summarized in Figs 4 and 5. Fig 4 shows the reputation scores obtained over multiple iterations, indicating how the scores evolve as the process progresses. The results demonstrate that the reputation mechanism effectively updates the scores based on the specified criteria, maintaining consistency and reliability. Fig 5 illustrates the time taken to calculate the reputation scores in each iteration. The time is recorded in milliseconds, providing a detailed view of the computational overhead introduced by the reputation mechanism. The test results indicate that while there is an increase in computation time with the number of iterations, the overhead remains manageable.

### ABFT protocol experiment

We evaluate the performance of Dumbo, the core BFT protocol integrated into our proposed anonymous communication system SRFACS. To demonstrate the advantages of our system, we compare it with two other widely used BFT protocols, PBFT (HTTP) and HoneyBadger, which serve as benchmarks in the experiment.

The experiments were performed on an Alienware x17 R1 laptop with an Intel(R) Core(TM) i7-11800H CPU @ 2.30GHz, 32GB of RAM, and running Windows 11 Professional Insider Preview. Following the methodology outlined in [24], we measured the latency of the Dumbo protocol for different byte sizes: 250, 500, 750, and 1000 bytes. PBFT (over HTTP) and HoneyBadger were included in the experiments to provide a comparative analysis of their performance against the SRFACS system using Dumbo.

#### Experimental results and analysis

**Fig 6:**
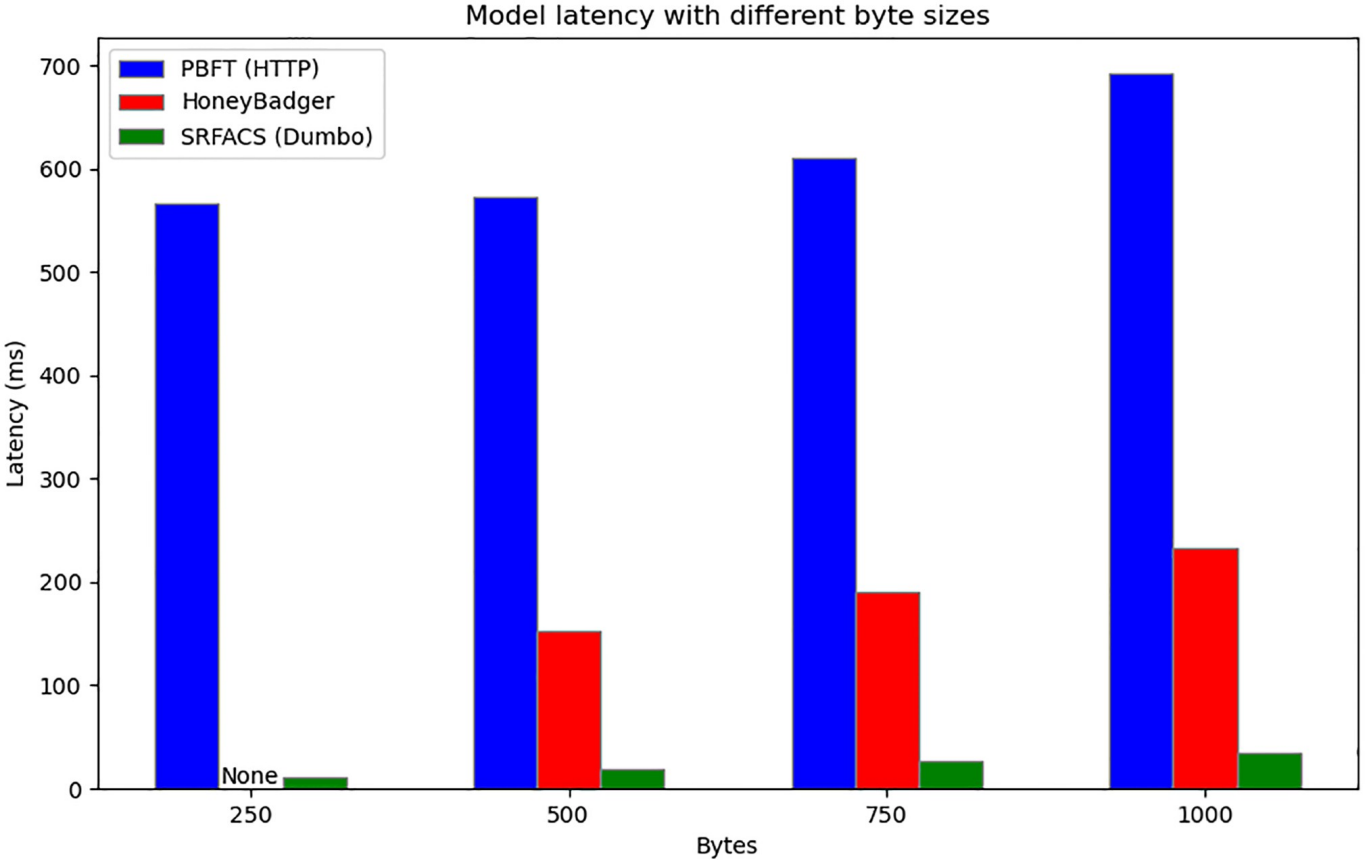
Latency comparison of PBFT (HTTP), HoneyBadger, and SRFACS (Dumbo) with different byte sizes.

**Fig 7:**
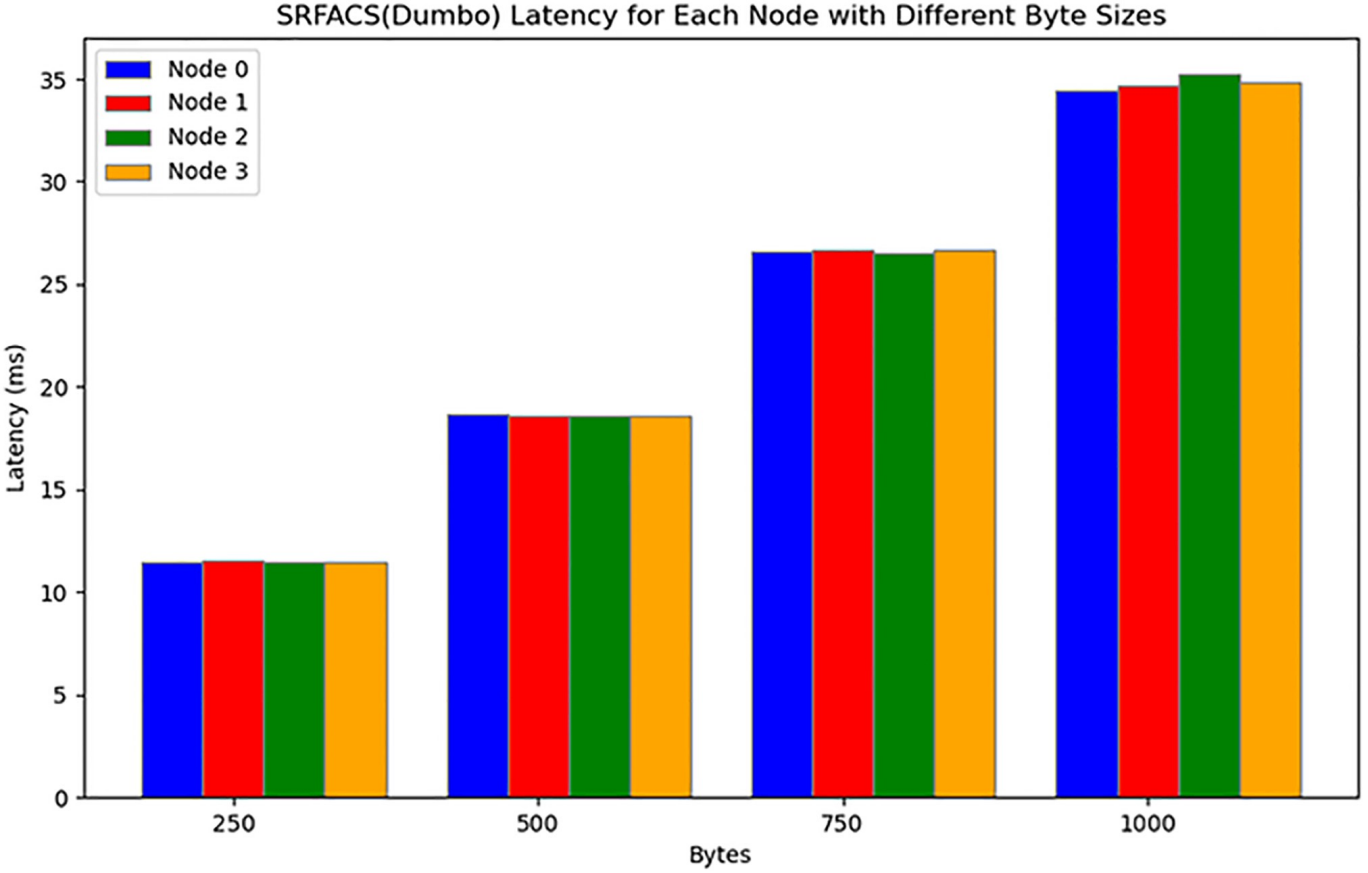
SRFACS(Dumbo) latency for each node with different byte sizes.

The results of the experiments are shown in Figs 6 and 7. Dumbo, as implemented in SRFACS, consistently exhibits the lowest latency across all byte sizes, significantly outperforming PBFT (HTTP) and HoneyBadger. This demonstrates that SRFACS, powered by Dumbo, can efficiently handle larger data volumes while maintaining low latency, making it highly suitable for anonymous communication systems. PBFT (HTTP) shows the highest latency, particularly as byte sizes increase, which reveals its limitations in environments that require higher throughput and lower latency. HoneyBadger performs better than PBFT (HTTP) but still cannot match the performance of Dumbo, especially as byte sizes grow. The results highlight the scalability and efficiency of SRFACS, which benefits from the integration of Dumbo.

In conclusion, the experimental results confirm that SRFACS, utilizing Dumbo as its core protocol, achieves significantly lower latency compared to the baseline protocols. The comparison with PBFT (HTTP) and HoneyBadger illustrates that SRFACS is optimized for performance, ensuring minimal latency even under increasing data loads. This efficiency and scalability make SRFACS an ideal solution for secure and anonymous communication in dynamic environments.

## Conclusion

We introduced the SRFACS framework aimed at enhancing the resilience and security of anonymous communications through the integration of advanced cryptographic techniques and network management strategies. Our framework employs an asynchronous Byzantine fault tolerance (ABFT) protocol, a multi-signature process with public-key aggregation, and a leader change protocol enhanced by a reputation-based incentive mechanism. These elements collectively improve fault tolerance, enabling SRFACS to handle up to (*n* − 1)/3 faulty nodes effectively and maintain high performance and reliability even under adverse conditions.

The performance evaluation demonstrated that SRFACS effectively supports scalable deployments while maintaining system integrity and operational efficiency. The framework ensures operational integrity and reduces the risks of failure, making it well-suited for environments requiring high security and anonymity. Future work will focus on optimizing these cryptographic operations and extending SRFACS’s applicability to more diverse network scenarios to further enhance its fault tolerance and scalability.
